# GluN2A/ERK/CREB Signaling Pathway Involved in Electroacupuncture Regulating Hypothalamic-Pituitary-Adrenal Axis Hyperactivity

**DOI:** 10.3389/fnins.2021.703044

**Published:** 2021-09-30

**Authors:** Yu Wang, Jing Han, Jing Zhu, Mizhen Zhang, Minda Ju, Yueshan Du, Zhanzhuang Tian

**Affiliations:** ^1^State Key Laboratory of Medical Neurobiology, Department of Integrative Medicine and Neurobiology, Brain Science Collaborative Innovation Center, School of Basic Medical Sciences, Institutes of Brain Science, Fudan Institutes of Integrative Medicine, Fudan University, Shanghai, China; ^2^Department of Anatomy, School of Basic Medicine, Shanghai University of Traditional Chinese Medicine, Shanghai, China

**Keywords:** GluN2A/ERK/CREB signaling pathway, HPA axis, electroacupuncture, CRH, surgical trauma

## Abstract

The hyperactivity of the hypothalamic-pituitary-adrenal (HPA) axis caused by stress will inevitably disrupt the homeostasis of the neuroendocrine system and damage physiological functions. It has been demonstrated that electroacupuncture (EA) can modulate HPA axis hyperactivity during the perioperative period. As the initiating factor of the HPA axis, hypothalamic corticotrophin-releasing hormone (CRH) is the critical molecule affected by EA. However, the mechanism by which EA reduces CRH synthesis and secretion remains unclear. Activated N-methyl-D-aspartate receptor (NMDAR) has been linked to over-secretion of hypothalamic CRH induced by stress. To determine whether NMDAR is involved in EA regulating the over-expression of CRH, a surgical model of partial hepatectomy (HT) was established in our experiment. The effect of EA on hypothalamic NMDAR expression in HT mice was examined. Then, we investigated whether the extracellular regulated protein kinases (ERK)/cyclic adenosine monophosphate response element-binding protein (CREB) signaling pathway mediated by NMDAR was involved in EA regulating HPA axis hyperactivity. It was found that surgery enhanced the expression of hypothalamic CRH and caused HPA axis hyperactivity. Intriguingly, EA effectively suppressed the expression of CRH and decreased the activation of GluN2A (NMDAR subunit), ERK, and CREB in HT mice. GluN2A, ERK, and CREB antagonists had similar effects on normalizing the expression of CRH and HPA axis function compared with EA. Our findings suggested that surgery enhanced the activation of the hypothalamic GluN2A/ERK/CREB signaling pathway, thus promoting the synthesis and secretion of CRH. EA suppressed the phosphorylation of GluN2A, ERK, and CREB in mice that had undergone surgery, indicating that the GluN2A/ERK/CREB signaling pathway was involved in EA alleviating HPA axis hyperactivity.

## Graphical Abstract

Surgery induced HPA axis hyperactivity, while EA intervention inhibited the activation of hypothalamic GluN2A/ERK/CREB signaling pathway, thereby reducing the synthesis and secretion of CRH, alleviating the hyperactivity of the HPA axis after surgery.

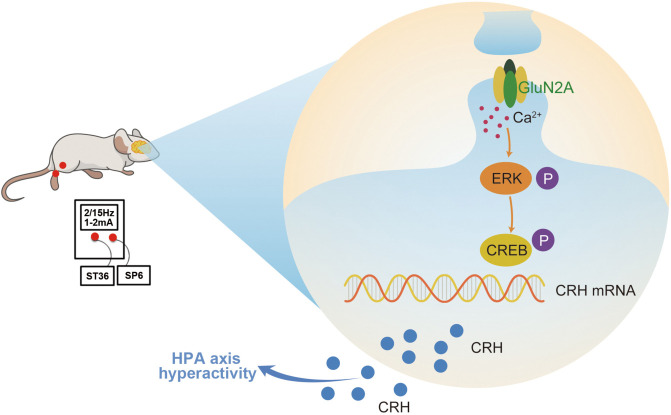

## Introduction

The hypothalamic-pituitary-adrenal (HPA) axis is defined as an indispensable neuroendocrine axis that involves stress and immunity response (Herman et al., 2016; Tapp et al., 2019). With advances in modern medical technology, surgery plays an increasingly important role in treating diseases. However, exposure to anesthesia, trauma, and pain during surgical operation will inevitably cause HPA axis hyperactivity (El-Sibai et al., 2017; Khawandanah et al., 2019), which is characterized by the excessive release of hypothalamic corticotropin-releasing hormone (CRH), peripheral adrenocorticotropic hormone (ACTH), and corticosterone (CORT) (Wu et al., 2020). It has been reported that HPA axis hyperactivity is closely associated with abnormal metabolism, neuroinflammation, affective disorders, and mental illness, and it seriously hinders recovery from surgical trauma and diminishes the quality of life of patients (DeMorrow, 2018; Kim, 2018; Ahmad et al., 2021). Therefore, it is an urgent task to explore the mechanism of HPA axis hyperactivity caused by surgical trauma and find an effective treatment to alleviate perioperative HPA axis dysfunction.

Hypothalamic CRH neurons, the key component of the HPA axis, are densely innervated by glutamatergic and GABAergic nerve projections (Wittmann et al., 2005; Maguire, 2014; Yuan et al., 2019). The functional N-methyl-D-aspartic acid receptor (NMDAR), a member of the glutamate receptor (GluR) family of ligand-gated ion channels, is a heterotetramer composed of GluN1 and GluN2 (A-D) or GluN3 (A-B) subunits (Hansen et al., 2018). Studies have highlighted the essential roles of NMDAR in brain signal conduction and nerve function (Yang et al., 2018; Zoodsma et al., 2020). Previous reports indicated that NMDAR is involved in various stress responses and plays a vital role in coordinating HPA axis activity (Collison et al., 2018; Lin et al., 2019). Activation of NMDAR alters synaptic plasticity and evokes calcium spikes in the hypothalamic paraventricular nucleus (PVN) neurons (Bains and Ferguson, 1999). Additionally, chronic unpredictable mild stress (CUMS) has been found to enhance NMDAR expression in the hypothalamus, while the NMDAR antagonist downregulates the frequency of miniature excitatory postsynaptic currents (mEPSCs) of CRH neurons (Zhou et al., 2018). Activated NMDAR contributes to the phosphorylation of ERK and CREB, which has been implicated in protein translation (Lai et al., 2014; Ko et al., 2018). CREB binds to c-AMP response element (CRE) in the promoter region of CRH after phosphorylation at the Ser133 site and then activates the transcription of CRH (Jeanneteau et al., 2012). Therefore, the NMDAR/ERK/CREB signaling pathway may be a potential therapeutic target for CRH over-expression and HPA axis hyperactivity during the perioperative period.

Electroacupuncture (EA) has been validated as a complementary and alternative therapeutic method to treat endocrine disorders including HPA axis hyperactivity (Zhu et al., 2017), polycystic ovary syndrome (PCOS) (Peng et al., 2020), and diabetes (Protto et al., 2019). A clinical study found that EA could reduce the excessive release of glucocorticoids and alleviate gastrointestinal discomfort caused by trauma (Geng et al., 2018). Experimental study indicated that EA suppresses the synthesis of hypothalamic CRH, peripheral ACTH, and CORT in rats under simulated weightlessness (Zhang et al., 2015). Our previous study demonstrated that EA at *Zusanli* (ST36) and *Sanyinjiao* (SP6) acupoints could reduce hypothalamic CRH expression in surgically traumatized rats, thus normalizing the HPA axis function (Zhang et al., 2021). However, the therapeutic mechanism of EA in alleviating HPA axis hyperactivity is still poorly understood.

Observational studies showed that EA downregulated the expression of NMDAR in rat hippocampus (Zhang et al., 2016). The level of GluN2A was markedly enhanced 2 days after spinal cord injury and then significantly reduced after EA treatment (Tu et al., 2017). Suppression of the phosphorylation of ERK following ischemic stroke stress by EA may provide neuroprotection against cerebral ischemic damage (Xing et al., 2018). We hypothesized that hypothalamic NMDAR and its downstream ERK/CREB signaling pathway may be affected by surgery and involved in the process of EA modulating HPA axis hyperactivity. Our results verified that EA reduced the activation of the GluN2A/ERK/CREB signaling pathway and ameliorated the over-secretion of CRH, thereby alleviating HPA axis hyperactivity in hepatectomy (HT) mice. This study provides a better understanding of the pathophysiological and molecular mechanisms of HPA axis hyperactivity induced by surgery and provides a new theoretical and practical basis for the clinical promotion of EA.

## Materials and Methods

### Experimental Animals

Male C57BL/6J mice weighing 18–22 g (Slack Laboratory Animal Center, Shanghai Branch of the Chinese Academy of Sciences, Shanghai, China) were kept at 22–24°C, with a 12/12 h circadian rhythm and *ad libitum* access to food and water. All of the experiments followed the National Institutes of Health Guide for the Care and Use of Laboratory Animals (NIH Publication No. 8023, revised 1978) and were approved by the Animal Care and Use Research Ethical Standards of Fudan University (Shanghai, China).

### Hepatectomy Model Preparation and Electroacupuncture Treatment

Animals were randomized into three groups after 1 week of adaptation: normal control (NC) group, hepatectomy (HT) group, and hepatectomy + EA (HT + EA) group (*n* = 6 in each group). Mice in each experimental group were habituated to the self-made fixing devices (50 mL centrifuge tubes with enough holes to make sure mice could breathe normally and facilitate EA stimulation) once a day for three consecutive days before surgery. After the habituation, all mice were anesthetized using Avertin (350 mg/kg, ip). Mice in the NC group were dealt no extra operation, and partial hepatectomy was performed in the HT and HT + EA groups as previously described (Zhu et al., 2017). In brief, an incision was made along the midline of the abdomen, and approximately 20% of the left liver lobe was removed.

Based on our preliminary experiments (Zhu et al., 2016), EA was performed 24 h before and immediately after surgery. Sterilized stainless steel needles (0.22 mm in diameter and 13 mm in length; *Huatuo*, Suzhou, China) were unilaterally inserted into *Zusanli* (ST36, 2 mm below the fibular head at the posterolateral knee of hind limbs) (Zhao et al., 2018) and *Sanyinjiao* (SP6, 5 mm above the tip of the medial malleolus on the posterior border of the tibia) (Wu et al., 2018) to a depth of 1 mm. Then, the needle handles were connected to the HANS Acupoint Nerve Stimulator (LH202H, Beijing, China), and a stimulation (2/15 Hz; frequency: 2 Hz 1.05 s, 15 Hz 2.85 s; 2 mA intensity) was applied for 30 min (within the period of 8:00–10:00 AM). Mice in the HT + EA group were restrained in the homemade fixing devices and were kept awake during EA treatment to avoid side effects from anesthetic. Meanwhile, mice in the NC and HT groups were also fixed in the restraining devices without needle insertion. At the end of the experiments (24 h after HT), animals in each group were decapitated after the administration of Avertin. The peripheral blood and hypothalamus were then collected on ice.

### Drug Administration

The drug delivery system was designed by RWD Life Science (RWD, Shenzhen, China). Cannula implantation was performed 10 days before drug administration. The administration location was ± 0.2 mm lateral, 0.6 mm rostral, and 4.5 mm ventral to the bregma (Zhang et al., 2020). The separated groups of mice were administrated NMDA (M3262, Sigma, United States; an agonist of GluN2A-containing NMDA receptors, 0.4 nmol/μL, 0.5 μL/side) (Carvalho-Netto et al., 2009), PEAQX (HY-12294A, Med Chem Express, China; a preferential antagonist of GluN2A-containing NMDA receptors, 1 μg/μL, 0.25 μL/side) (Liu X.Y. et al., 2020), MEK1/2 Inhibitor IV (444967, Sigma, United States; an inhibitor of ERK, 0.1 nmol/μL, 0.5 μL/side) (Klein et al., 2006), 666-15 (HY-101120, Med Chem Express, China; an antagonist of CREB, 0.1 nmol/μL, 0.5 μL/side) (Yu et al., 2020), or normal saline into the bilateral hypothalamus (4–6 animals in each group) for functional research. Drugs were dissolved in dimethyl sulfoxide (DMSO) and diluted in 0.9% sterile normal saline (NS). Mice were randomly assigned into the following groups: (1) normal saline (NS), NMDA, NMDA + EA, and EA group; (2) NS, HT + NS, HT + PEAQX, and HT + EA group; (3) NS, HT + NS, HT + MEK, and HT + EA group; (4) NS, HT + NS, HT + 666-15, and HT + EA group. Drugs or normal saline were injected on the same day as the EA treatment (24 h before and immediately after the surgery at 8:00–10:00 a.m.) at a rate of 0.1 μL/min. All mice were anesthetized and sacrificed 24 h after the last drug administration. The peripheral blood and hypothalamus tissues were harvested from the experimental animals on ice.

### Enzyme Linked Immunosorbent Assay

Peripheral blood samples were collected 24 h after surgery and centrifuged at 3,000 rpm for 30 min after standing at room temperature for 1 h. Serum levels of ACTH (BPE20424, Lengton Biological Technology, China) and CORT (BPE20382, Lengton Biological Technology, China) were analyzed using an ELISA kit according to the manufacturer’s instructions. The absorbance (OD) was measured at 450 nm. The hormone levels in the samples were calculated according to the standard data curve.

### RT-PCR Analysis

Total RNA was extracted with TRIzol Reagent (204204, Thermo Fisher Scientific, Carlsbad, United States) from the hypothalamus according to the manufacturer’s instructions, and approximately 2 μg of total RNA was reverse-transcribed to cDNA (RR036A, Prime Script^TM^ RT Master Mix, TaKaRa, Japan). Real-time quantitative PCR amplification was performed to detect the expression of GluN1, GluN2A, and GluN2B mRNA using a TB Green Kit (TB Green^®^ Premix Ex Taq^TM^ TaKaRa, Japan). The primer sequences were as follows: CRH (Forward: GGG AGT CAT CCA GTT GTT T; Reverse: GAG CTT ACA CAT TTC GTC CTA), GluN1 (Forward: AGA GCC CGA CCC TAA AAA GAA; Reverse: CCC TCC TCC CTC TCA ATA GC), GluN2A (Forward: AGA CCT TAG CAG GCC CTC TC; Reverse: CTC TTG CTG TCC TCC AGA CC), GluN2B (Forward: GGC ACA CAG GAC ACA TAA ACC; Reverse: ACC AGG AAG GCA AGA AGC A), GAPDH (Forward: AAA TGG TGA AGG TCG GTGTG; Reverse: AGG TCA ATG AAG GGG TCG TT). Levels of related mRNAs were calculated using the 2−^ΔΔ*CT*^ method and normalized to those of GAPDH mRNA.

### Western Blot Analysis

The hypothalamus tissue (30 mg) was lysed with RIPA buffer (P0013B, Beyotime, China) containing PMSF (ST506, Beyotime, China) and Halt^TM^ Phosphatase Inhibitor Cocktail (78427, Thermo Fisher Scientific, Carlsbad, United States) (100:1:1). An equal amount of the protein sample (40 μg) was subjected to 6 or 12% sodium dodecyl sulfate-polyacrylamide gel electrophoresis (SDS-PAGE), and the segregated protein was transferred onto a polyvinylidene fluoride (PVDF) membrane (Millipore, Darmstadt, Germany). The membrane was then blocked in TBST buffer containing 5% (w/v) non-fat milk for 2 h at RT and incubated with primary antibodies against CRH (ab184238, anti-rabbit, 1:800; Abcam; Cambridge, United Kingdom), phosphor-Y1325 GluN2A (ab106590, anti-rabbit, 1:1,000; Abcam), GluN2A (ab203197, anti-rabbit, 1:1,000; Abcam), phosphor-ERK1/2 (4370, anti-rabbit, 1:1,000; Cell Signaling Technology; Danvers, MA, United States), Erk1/2 (4695, anti-rabbit, 1:1,000; Cell Signaling Technology), Phosphor-CREB Ser133 (9198S, anti-rabbit,1:1,000; Cell Signaling Technology), CREB (12208-1-AP, anti-rabbit, 1:1,000; Proteintech; United States) and β-tubulin (10094-1-AP, anti-mouse,1:10,000, Proteintech; United States) overnight at 4°C. Subsequently, the membrane was incubated for 2 h at RT with Horseradish Peroxidase (HRP)-conjugated secondary antibody (Goat anti-Rabbit IgG Secondary Antibody HRP conjugated, Signalway Antibody LLC, United States). The protein bands were visualized by enhanced chemiluminescence (ECL kit, WBKLS0500, Millipore, Darmstadt, Germany). The relative optical density of protein bands was quantified using Quantity One^®^ software (version 4.0.3) from Bio-Rad (Hercules, CA, United States). The band densities of target proteins were counted separately. Band densities for the phosphorylation proteins were normalized to the corresponding total proteins, and the density of the CRH protein band was normalized to corresponding β-tubulin for each sample.

### Immunofluorescence

Mice in each group were deeply anesthetized with Avertin (350 mg/kg, ip) 24 h after HT, and perfused with 0.1 M phosphate-buffered saline (PBS, pH 7.4) followed by 4% paraformaldehyde (PFA). Brains were dissected from the skulls carefully, post-fixed in PFA overnight, and then dehydrated in 20 and 30% sucrose solution at 4°C. After dehydration, the whole brain was sliced into 25 μm thickness using a cryostat microtome. Slices located in the 0.7–1.06 mm behind the bregma were selected. Each slice was washed three times with phosphate-buffered saline (PBS) for 10 min, blocked for 1 h with blocking solution (P0260, Beyotime, China), and then incubated overnight at 4°C with primary antibodies against CRH (ab8901, anti-rabbit, 1:50; Abcam), GluN2A (NBP2-22405, anti-mouse, 1:100; Novus), and phosphor-ERK1/2 (4370, anti-rabbit, 1:200; Cell Signaling Technology). Next, each slice was incubated in Alexa Fluor 488 (A-21206, 1:1,000; Thermo Fisher Scientific) or Alexa Fluor^®^ 594 (ab150080, 1:1,000; Abcam) conjugated secondary antibodies in the dark for 2 h at RT. The sections were then observed with an Olympus FV-1000 laser confocal scanning microscope (Olympus Corporation, Shinjuku, Tokyo, Japan). For quantification of immunostained cells, CRH (+), CRH and GluN2A co-labeled, and pERK (+) cells were counted in the area of 400 μm^2^ beside the third ventricle (3V). An observer, blind to the treatments, recorded the data. The number of positive cells in the dedicated area was quantified using ImageJ software (version 1.8.0; National Institutes of Health, Bethesda, MD, United States).

### Immunohistochemistry

Under anesthesia with Avertin, all mice were perfused with 0.1 M PBS followed by 4% PFA 24 h after HT. The brain tissue was post-fixed in 4% PFA at 4°C overnight and cut into 6-μm-thick sections. The sections were deparaffinized and rehydrated in descending concentrations of ethanol, and then they were treated with 0.01 M citric acid buffer (15 min, 98°C) in a microwave oven for antigen retrieval and washed with ddH_2_O 3 × 5 min. The specimens were incubated in 0.3% H_2_O_2_ in 0.1 M PBS for 20 min to inactivate endogenous peroxidase activity followed by diluted anti-pGluN2A antibody (ab16646, 1:50; Abcam) for 2 h at 37°C. The slices were then incubated with the biotin-labeled secondary antibody for 30 min at RT. Diaminobenzidine (DAB) chromogen (AR-1022, Boster, Wuhan, China) was added to visualize the immune reaction. After being counterstained with hematoxylin, the specimens were dehydrated and mounted, and then observed with an Olympus microscope. Immunohistochemistry-stained cells that appeared yellow were positive. The pGluN2A positive cells in the area of 400 μm^2^ beside 3V were counted using ImageJ software (version 1.8.0; NIH).

### Statistical Analysis

Statistical analyses were performed using GraphPad Prism 7 software (GraphPad Software Inc., San Diego, CA, United States). Data were presented as the mean ± standard error of the mean (SEM). One-way analysis of variance (ANOVA) was used to compare the differences between two groups. *P <* 0.05 was considered statistically significant for all of the analyses.

## Results

### Electroacupuncture Ameliorates Surgery-Induced Hyperactivity of the Hypothalamic-Pituitary-Adrenal Axis

**FIGURE 1:**
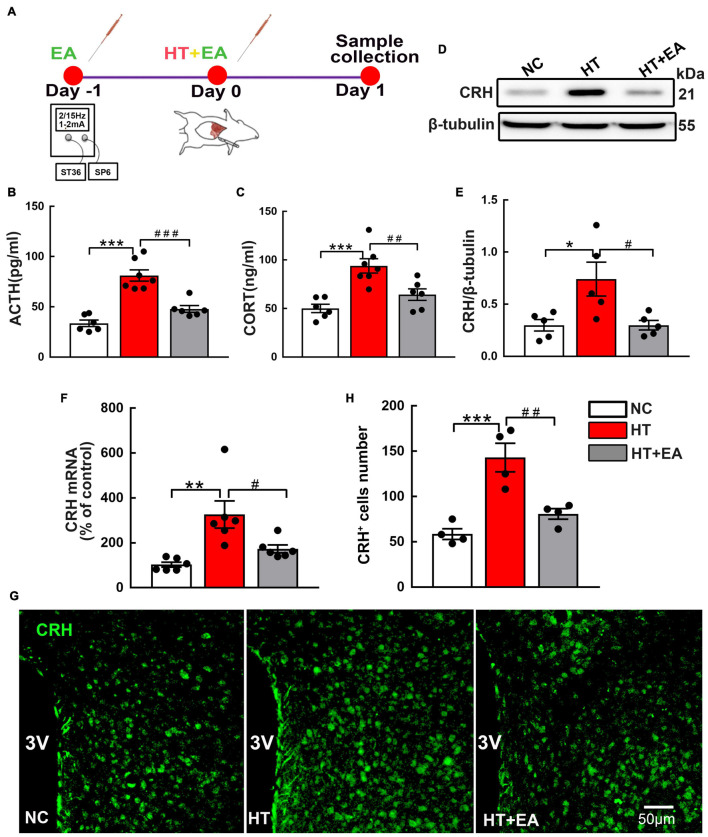
EA ameliorates the HPA axis hyperactivity induced by surgery. **(A)** Timeline of experimental protocol. **(B)** ACTH and **(C)** CORT in the peripheral serum of mice in the NC, HT, and HT + EA groups (*n* = 6 for each group). **(D,E)** CRH protein and **(F)** mRNA expression in the hypothalamus. The CRH protein bands were normalized to β-tubulin. **(G,H)** Representative immunofluorescence images and quantification for CRH-positive cells in the PVN (*n* = 4 for each group). Data are expressed as mean ± SEM. *vs. NC group (^∗^*p* < 0.05, ^∗∗^*p* < 0.01, ^∗∗∗^*p* < 0.001); #vs. HT group (^#^*p* < 0.05, ^##^*p* < 0.01, ^###^*p* < 0.001). One-way analysis of variance (ANOVA). CRH, Corticotrophin-releasing hormone; ACTH, Adrenocorticotropic hormone; CORT, Corticosterone; PVN, Paraventricular nucleus.

The serum ACTH and CORT concentrations were measured as indices of HPA axis activity. The experimental timeline is shown in Figure 1A. ELISA results showed that ACTH and CORT levels in the HT group were markedly higher than those in the NC group (*p <* 0.001). However, hormone levels in the HT + EA group were significantly lower than those in the HT group (*p* < 0.001 for ACTH, *p* < 0.01 for CORT) (Figures 1B,C). Additionally, the expressions of hypothalamic CRH protein and mRNA in the HT group were significantly increased compared with those in the NC group (*p* < 0.05 for protein, *p* < 0.01 for mRNA), whereas they were decreased notably in the HT + EA group (*p* < 0.05) (Figures 1D–F). Consistent with the results of western blot and RT-PCR, immunofluorescence staining showed that surgery promoted the number of PVN CRH-positive cells compared with NC cells (*p* < 0.001), whereas the upregulation of CRH-positive cells was alleviated considerably by EA treatment (*p <* 0.01) (Figures 1G,H). These results indicated that trauma-induced HPA axis hyperactivity was relieved by EA, which means EA had a prominent therapeutic effect on regulating HPA axis dysfunction.

### Electroacupuncture Attenuates Expression and Phosphorylation of the Hypothalamic GluN2A-NMDA Receptor After Surgery

**FIGURE 2:**
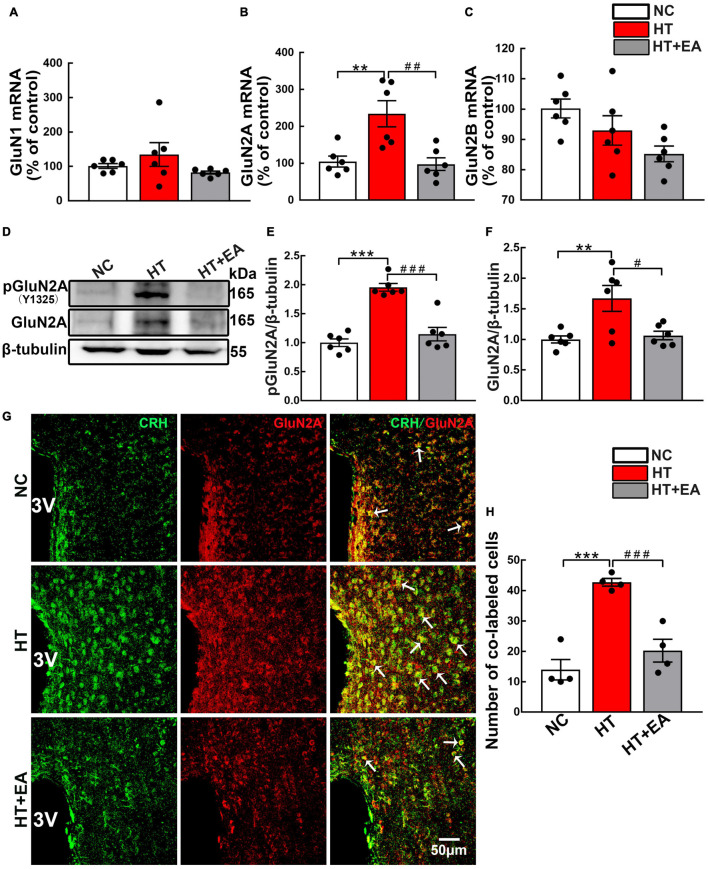
EA reduces hypothalamic GluN2A expression after surgery. Quantification of hypothalamic **(A)** GluN1, **(B)** GluN2A, and **(C)** GluN2B mRNA levels in the NC, HT, and HT + EA groups (*n* = 6 for each group). **(D–F)** Expression of phosphorylated GluN2A (pGluN2A) and total GluN2A protein in the hypothalamus was analyzed by western blot (*n* = 6 for each group). **(G,H)** Representative images and quantification for Co-labeled CRH and GluN2A-positive cells in the PVN (*n* = 4 for each group). Data are expressed as mean ± SEM. *vs. NC group (**p < 0.01, ***p < 0.001); #vs. HT group (^#^*p* < 0.05, ^##^*p* < 0.01, ^###^*p* < 0.001). One-way analysis of variance (ANOVA).

To determine the potential involvement of NMDAR in HPA axis hyperactivity, the content and phosphorylation of NMDAR in the hypothalamus were examined. RT-PCR results revealed that hypothalamic GluN2A mRNA expression was markedly upregulated in the HT group (*p* < 0.01) (Figure 2B) and notably decreased in the HT + EA group (*p* < 0.01). Intriguingly, neither HT nor EA affected the mRNA levels of GluN1 and GluN2B subunits in the hypothalamus (*p* > 0.05) (Figures 2A,C). Furthermore, surgery increased the expression of phosphorylated (*p* < 0.001) and total GluN2A (*p* < 0.01) protein in the hypothalamus (Figures 2D–F), whereas EA notably decreased the level of phosphorylated (*p* < 0.001) and total GluN2A (*p* < 0.05) protein. Surgical trauma consistently caused a significant increase in the number of PVN phosphorylated GluN2A-positive cells (*p* < 0.05), which was alleviated by EA treatment (*p* < 0.05) (Supplementary Materials). Immunofluorescence staining revealed that CRH and GluN2A positive cells were widely co-labeled in the PVN of surgical trauma mice. After EA treatment, the number of co-localization cells reduced notably (*p* < 0.001) (Figures 2G,H). Taken together, the results above indicated that EA downregulated the expression and activation of GluN2A in the hypothalamus.

### Electroacupuncture Reduces the Over-Expression of Hypothalamic Corticotrophin-Releasing Hormone in NMDA-Administered Mice

**FIGURE 3:**
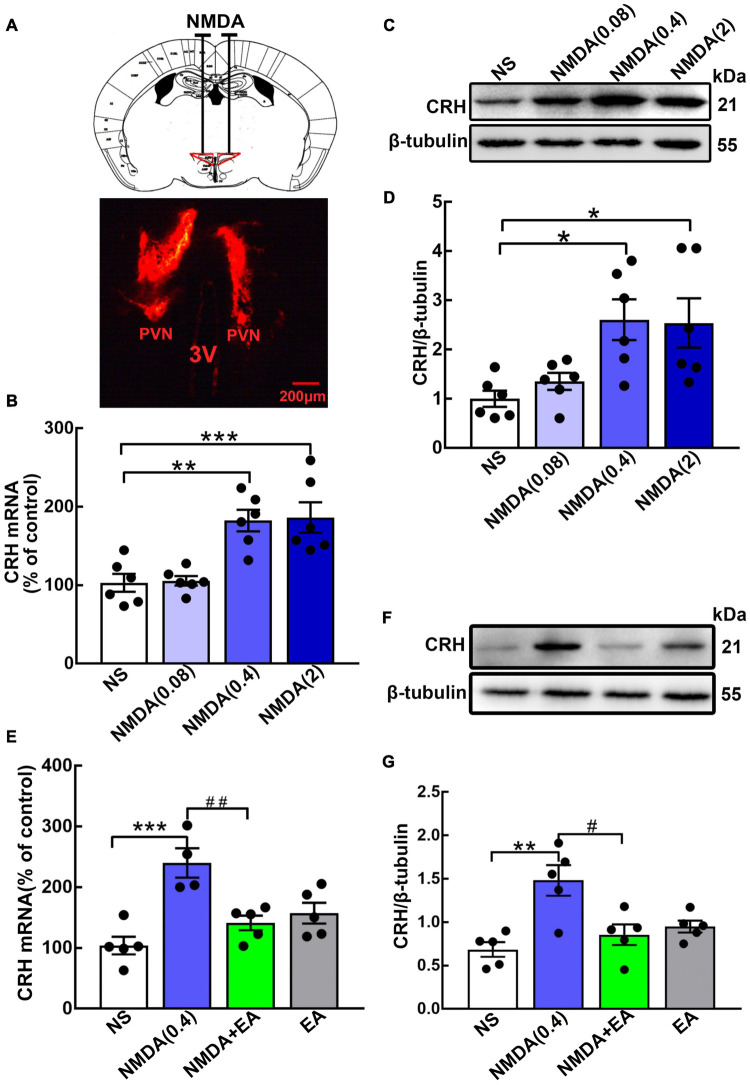
Hypothalamic CRH expression was downregulated after EA treatment in NMDA-administered mice. **(A)** Cannula implantation was performed and fluorescent dye (DiIC18) was injected into the hypothalamus through the cannula to determine the accuracy of drug injection (Administration location: AP 0.6 mm, ML ± 0.2 mm, DV 4.5 mm). For the GluN2A agonist, different doses of NMDA (0.08 nmol/μL, 0.4 nmol/μL, and 2 nmol/μL) were injected into the bilateral hypothalamus. **(B)** Quantification of hypothalamic CRH mRNA. **(C,D)** Representative WB images and quantification for CRH protein (*n* = 6 for each group) 24 h after NMDA injection. Quantification of CRH **(E)** mRNA and **(F,G)** protein in the NS, NMDA, NMDA + EA, and EA groups (n = 5 for each group). Data are expressed as mean ± SEM. *vs. NS group (*p < 0.05, **p < 0.01, ***p < 0.001); #vs. NMDA group (^#^*p* < 0.05, ^##^*p* < 0.01).

Different concentrations of NMDA were administered to the hypothalamus to observe the effect of the NMDAR agonist on CRH expression (Figure 3A). Here, we found that the level of hypothalamic CRH was increased dose-dependently by NMDA. The hypothalamic CRH mRNA increased significantly after the injection of NMDA at the concentrations of 0.4 nmol/μL (*p* < 0.01) and 2 nmol/μL (*p* < 0.001), but it was not changed by normal saline or 0.08 nmol/μL NMDA (Figure 3B). Then we further evaluated CRH protein expression in the hypothalamus, and the western blot results showed that CRH protein was upregulated after the administration of NMDA (*p* < 0.05 for 0.4 nmol/μL and 2 nmol/μL) (Figures 3C,D).

Mice were given EA treatment after the administration of NMDA (0.4 nmol/μL). Compared with the NS group, hypothalamic CRH was upregulated in the NMDA group, whereas EA considerably decreased the expression of CRH in mice administered with NMDA (*p* < 0.01 for mRNA, *p* < 0.05 for protein) (Figures 3E–G). These results suggested that NMDA increased the level of CRH, and EA may have decreased the activation of GluN2A, thus reducing the synthesis and secretion of CRH.

### Extracellular Regulated Protein Kinases/Cyclic Adenosine Monophosphate Response Element-Binding Protein Signaling Pathway

**FIGURE 4:**
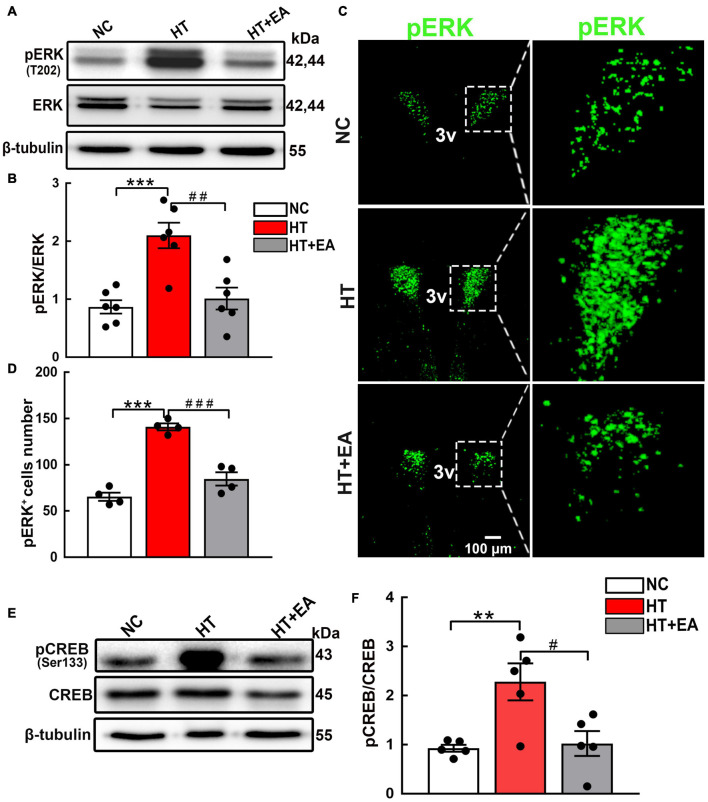
EA inhibits the phosphorylation of hypothalamic ERK and CREB. **(A,B)** Expression of hypothalamic phosphorylated ERK (pERK) and total ERK proteins in the NC, HT, and HT + EA groups (n = 6 for each group). **(C,D)** Immunofluorescence representative images and quantitative analysis of pERK-positive cells in the PVN (*n* = 4 for each group). **(E,F)** Expression of hypothalamus phosphorylated CREB and total CREB proteins (*n* = 5 for each group). The pERK and pCREB protein bands were normalized to ERK or CREB. Data are expressed as mean ± SEM. *vs. NC group (**p < 0.01, ***p < 0.001); #vs. HT group (^#^*p* < 0.05, ^##^*p* < 0.01, ^###^*p* < 0.001). ERK, Extracellular regulated protein kinases; CREB, cAMP-response element binding protein.

Having verified that the phosphorylation of hypothalamic GluN2A was upregulated in mice that underwent surgery, the change in the ERK/CREB signaling pathway mediated by GluN2A was detected. The level of hypothalamic phosphorylated ERK, but not ERK, increased significantly after surgical trauma (*p* < 0.001), and EA reduced ERK phosphorylation notably (*p* < 0.01) (Figures 4A,B). Additionally, the confocal images clearly demonstrated that EA reduced the number of pERK-positive cells in the PVN (*p* < 0.001) (Figures 4C,D). Hypothalamic CREB phosphorylation protein increased after surgery (*p* < 0.01), whereas EA effectively alleviated CREB phosphorylation (*p* < 0.05) (Figures 4E,F). Therefore, we concluded that surgical trauma caused activation of hypothalamic GluN2A and its downstream ERK/CREB signaling pathway, and EA alleviated the phosphorylation of the GluN2A/ERK/CREB signaling pathway.

### GluN2A/ERK/CREB Signaling Pathway Is Involved in the Process of Electroacupuncture Attenuating Hypothalamic-Pituitary-Adrenal Axis Hyperactivity

**FIGURE 5:**
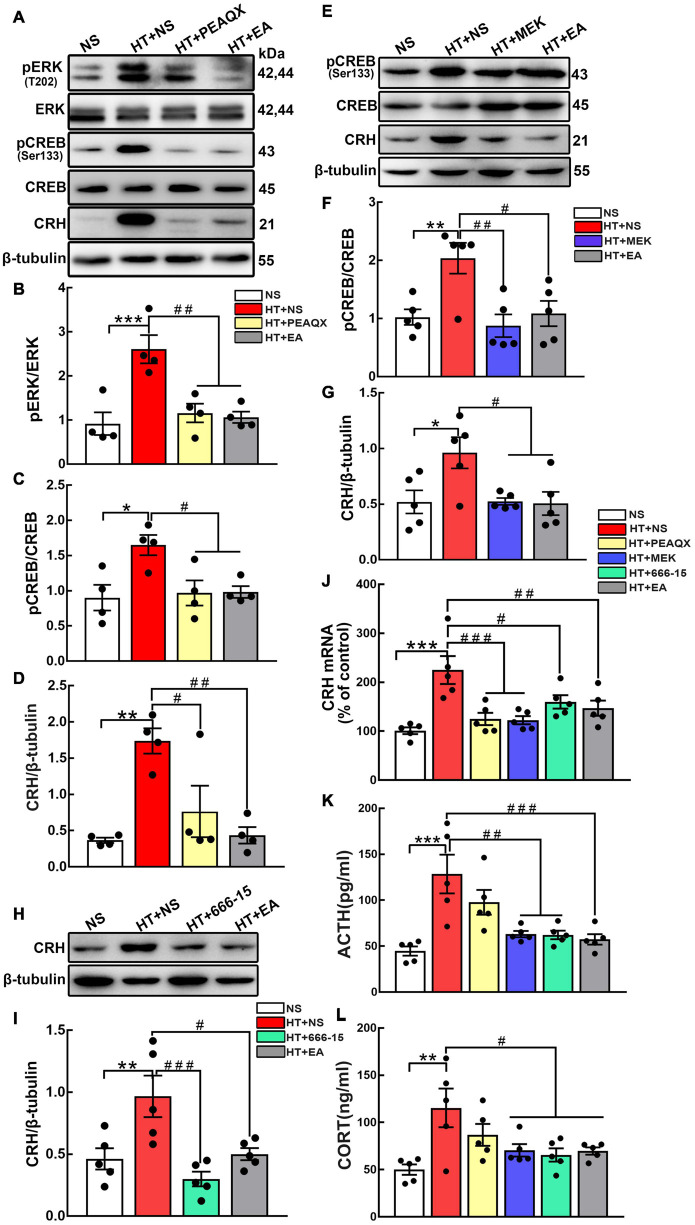
The GluN2A, ERK, and CREB antagonist reverses HPA axis hyperactivity induced by surgery. The GluN2A antagonist PEAQX (1 μg/μL, 0.25 μL/side) was administered to both sides of the hypothalamus. Hypothalamus was dissected 24 h after the PEAQX administration. **(A–D)** Representative bands and quantification of hypothalamic pERK, pCREB, and CRH protein among the NS, HT + NS, HT + PEAQX, and HT + EA groups (*n* = 4 for each group). The ERK inhibitor MEK1/2 Inhibitor IV (0.1 nmol/μL, 0.5 μL/side) was administered stereotaxically into the hypothalamus. **(E–G)** Representative bands and quantification of pCREB and CRH protein in the NS, HT + NS, HT + MEK, and HT + EA groups (*n* = 5 for each group). 666-15 (an antagonist of CREB, 0.1 nmol/μL, 0.5 μL/side) was administrated to investigate the relationship between CREB and HPA axis. **(H,I)** Western Blot analysis of hypothalamic CRH protein in the NS, HT + NS, HT + 666-15, and HT + EA groups (*n* = 5 for each group). **(J)** Relative hypothalamic CRH mRNA expression after surgical trauma and drugs administration (*n* = 5 for each group). **(K,L)** ACTH and CORT levels in the peripheral serum of mice (*n* = 5 for each group). Data are expressed as mean ± SEM. *vs. NS group (*p < 0.05, **p < 0.01, ***p < 0.001); #vs. HT group (^#^*p* < 0.05, ^##^*p* < 0.01, ^###^*p* < 0.001).

According to the results above, we hypothesized that the GluN2A/ERK/CREB signaling pathway is involved in the process of CRH synthesis and secretion. PEAQX (NVP-AAM077), aGluN2A specific antagonist, was administered stereotaxically into the hypothalamus. Western blot results showed that the GluN2A antagonist downregulated the level of pERK (*p* < 0.01) and pCREB (*p* < 0.05) (Figures 5A–C). More importantly, the GluN2A antagonist suppressed the expression of CRH compared with the HT + NS group (*p* < 0.05) (Figure 5D). Intriguingly, similar results were obtained after EA treatment. To investigate the role of hypothalamic pERK in HPA axis regulation, MEK1/2 Inhibitor IV, an ERK inhibitor, was injected into the hypothalamus of mice undergoing surgical trauma. Hypothalamic pCREB expression was found to decrease in the HT + MEK group compared with the HT + NS group (*p* < 0.01) (Figures 5E,F). Strikingly, western blot analysis showed that the inhibition of ERK impaired the over-expression of CRH (*p* < 0.05) (Figure 5G), and this effect was similar to EA intervention. Our results indicated that pERK was included in the signaling cascade for hypothalamic pCREB and CRH up-regulation. The potent and selective CREB inhibitor 666-15 was used to further identify the correlation between hypothalamic CREB and CRH. Compared with the HT + NS group, 666-15 administration significantly decreased the hypothalamic CRH protein level (*p* < 0.001) (Figures 5H,I). Moreover, CRH mRNA levels were found to downregulate in the HT + PEAQX (*p* < 0.001), HT + MEK (*p* < 0.001), HT + 666-15 (*p* < 0.05), and HT + EA (*p* < 0.01) groups in comparison with the HT + NS group (Figure 5J). Further analyses showed that the serum levels of ACTH and CORT were markedly downregulated after the administration of MEK1/2 Inhibitor IV or 666-15 (*p* < 0.01 for ACTH; *p* < 0.05 for CORT) (Figures 5K,L). Our results revealed the inhibition of the GluN2A/ERK/CREB signaling pathway alleviated HPA axis abnormality post-surgery. EA consistently reduced the ACTH and CORT concentrations significantly (*p* < 0.001 for ACTH; *p* < 0.05 for CORT), thereby alleviating HPA axis hyperactivity. Collectively, these results highlighted that EA treatment decreased the activation of the GluN2A/ERK/CREB signaling pathway and alleviated HPA axis dysfunction, and this effect was similar to that of GluN2A, ERK, and CREB antagonists.

## Discussion

In this study, we investigated the mechanism of the GluN2A/ERK/CREB signaling pathway’s involvement in EA alleviating HPA axis hyperactivity. Our results demonstrated that surgery caused activation of the hypothalamic GluN2A, ERK, and CREB, but EA could significantly ameliorate the activation of the GluN2A/ERK/CREB signaling pathway, and thereby suppress the expression of CRH and the hyperactivity of the HPA axis. Moreover, EA reversed the over-expression of CRH induced by NMDA administration in the hypothalamus. Furthermore, pharmacological inhibition of GluN2A, ERK and CREB lowered the level of hypothalamic CRH, which was consistent with the effect of EA. To the best of our knowledge, this is the first study to investigate the mechanism of the GluN2A/ERK/CREB signaling pathway in the regulation of HPA axis hyperactivity by EA.

The HPA axis serves as the hub connecting the nervous system and the endocrine system (Liyanarachchi et al., 2017; Cherian et al., 2019), so it plays a critical role in regulating hormone secretion, stress adaption, and energy metabolism (Joseph and Golden, 2017; de Oliveira et al., 2018). Unfortunately, its dysfunction induced by surgery, anesthesia, anxiety, and pain often results in endocrine disorders and immunosuppression (Kim, 2017; Tafet and Nemeroff, 2020). With the continuation of HPA axis hyperactivity, diseases such as diabetes (Miranda et al., 2016), high blood pressure (Muraca et al., 2021), and depression (Li et al., 2020) will inevitably appear. To simulate HPA axis hyperactivity in clinical situations, the HT mouse model was employed in the present study. Our previous research found that hyperfunction of the HPA axis can be seen 4 h after HT, and the phenomenon can last for up to 7 days (Zhu et al., 2017). Endocrine dysfunction, inflammation, and immunosuppression induced by HT simulated the symptoms of patients undergoing surgical trauma. Here, we found that the HPA axis was activated after surgery, as evidenced by the upregulated peripheral serum levels of ACTH and CORT. As the initiator of the HPA axis, hypothalamic CRH increased markedly after surgery.

Intriguingly, our study found that electrical stimulation at *Zusanli* and *Sanyinjiao* acupoints significantly lowered the expression of CRH and modulated HPA axis hyperactivity induced by surgery. EA regulates the HPA axis at multiple targets and levels, experimental studies demonstrated that EA could downregulate hypothalamic CRH and serum CORT levels in rats subjected to acute tail suspension stress (Zhang et al., 2015). Consistently, the expression of CRH mRNA in the hypothalamus and ACTH and CORT in plasma was decreased after EA treatment in CUMS rats (Le et al., 2016). The results above support the effectiveness of EA in regulating HPA axis dysfunction. The therapeutic effects of EA were determined by factors such as needle insertion location and the frequency of electrical stimulation. *Zusanli* and *Sanyinjiao* were found to be the most effective acupoints for HPA axis hyperactivity induced by cerebral ischemia-reperfusion injury (CI-RI) (Cai et al., 2009), traumatic stress (Zhang et al., 2021), and perinatal nicotine exposure stress (Liu et al., 2018). Our previous study revealed that non-acupoint intervention (NA, acupuncture needles were inserted into the ipsilateral root of the tail and electrical stimulation was applied) failed to ameliorate HPA axis dysfunction in trauma animals (Zhu et al., 2021). Better perioperative management could decrease the risk of HPA axis hyperactivity. Previous studies showed that anxiety and pain were associated with HPA axis dysfunction (Staniszewski et al., 2018; Fiksdal et al., 2019). As an effective intervention, acupuncture sessions before surgery has been found to relieve anxiety of patients (Shan et al., 2010; Tong et al., 2021). Besides, EA stimulation before gynecological laparoscopic surgery improved postoperative analgesia and alleviated postoperative side effects (Yao et al., 2015). The results above indicated that preoperative EA could reduce the potential harm and stress response caused by surgery.

NMDAR is widely distributed in the hypothalamus, especially in the paraventricular nucleus (PVN) and supraoptic nucleus (SON) (Ma et al., 2018). One experimental study suggested that GluN2A was markedly upregulated in postsynaptic spines 2 h after foot shock stress (Bonini et al., 2016). Significant pain behaviors and an increased level of GluN2A were shown in a rat model of chronic constriction injury (CCI) (Qian et al., 2019), suggesting that GluN2A participates in traumatic stress responses. Previous reports indicated that repeated stress escalated glutamatergic transmission and increased the NMDAR dependent burst-firing pattern in CRH positive neurons (Yuan et al., 2019). Our results showed that the number of GluN2A and CRH co-localization positive cells increased significantly in animals that underwent surgery. Furthermore, the level of hypothalamic CRH was increased dose-dependently by the GluN2A agonist. Stereotactic brain injection of GluN2A blocker (PEAQX) dramatically inhibited the phosphorylation of ERK and CREB, thereby reducing CRH synthesis and release. In summary, these results provide evidence that GluN2A plays an essential role in regulating the expression of hypothalamic CRH. We also noted that there was a tendency toward decreasing serum levels of ACTH and CORT after the administration of PEAQX. CRH is necessary for ACTH and CORT secretion, but there is a time lag before an increase or decrease in circulating ACTH and CORT levels (Spencer and Deak, 2017). Besides, it takes time for GluN2A antagonists to exert an effect on downstream ERK and CREB. We suspect it may take longer for the GluN2A antagonist in the hypothalamus to act on peripheral serum ACTH and CORT by inhibiting the GluN2A/ERK/CREB pathway.

Strikingly, the expression of GluN2A was significantly ameliorated after EA at *Zusanli* and *Sanyinjiao* acupoints, and the level of CRH was markedly downregulated in the NMDA + EA group. Moreover, the same therapeutic effect of reducing CRH synthesis was observed in the HT + EA group and HT + PEAQX group. The results indicated that electrical stimulation inhibited the over-expression of hypothalamic CRH caused by GluN2A activation. Here, we demonstrated that surgery induced the up-regulation of GluN2A, but GluN2A antagonist and EA intervention inhibited the features of GluN2A to downregulate CRH synthesis in the hypothalamus. Electrical stimulation at specific acupoints could distantly regulate the expression of neurotransmitters in the central nervous system (Zhang et al., 2016). Researches showed that peripheral EA stimulation significantly suppressed the expression of excitatory neurotransmitter glutamate in surgical trauma rats (Zhang et al., 2017). Besides, it has been reported that low-intensity EA at ST36 could induce c-Fos expression in vagal efferent neurons in the hindbrain, thus producing anti-inflammatory effects (Liu S. et al., 2020). The results indicated that the neuronal signals generated from the peripheral acupoints could be transmitted to the vagal circuit in the central nervous system. Additionally, researchers found that acupuncture could improve blood supply in the nerve system, thus reducing the levels of extracellular glutamate and NMDAR (Choi et al., 2010; Lee et al., 2010). The potential mechanism of EA influencing GluN2A in hypothalamic neurons may be related to neurotransmitters, the autonomic nervous system, and hypoxia/ischemia, although the specific mechanism requires further investigation.

ERK, a member of the mitogen-activated protein kinases (MAPK) family, is upstream of CREB and regulates phosphorylation of CREB. Researchers found that cocaine-induced phosphorylation of CREB (Ser133) can be blocked by ERK inhibitor (Sarkar et al., 2004; Somalwar et al., 2018). The ERK/CREB pathway has been demonstrated to play a critical role in stress response. Researchers revealed that the expression of hypothalamic pERK and pCREB was increased in the inflammatory pain model, but these changes were attenuated after EA treatment (Yen et al., 2019). In our data, the activation of the ERK/CREB pathway was increased after surgical trauma, but the administration of ERK inhibitor considerably decreased the expression of hypothalamic pCREB and CRH as well as serum levels of ACTH and CORT. Thus, we conclude that pERK is included in the proposed signaling cascade for NMDAR-dependent HPA hyperactivity.

The interaction between CREB and CRE is necessary for CRH transcriptional activation (Cope et al., 2014; Liu et al., 2011). A recent study showed that transfection with CREB plasmids escalates the expression of CRH (Tang et al., 2017). Our results showed that the CREB antagonist reduced hypothalamic CRH expression, which was accompanied by lower serum ACTH and CORT levels, indicating a close connection between CREB and the HPA axis. Intriguingly, EA ameliorated hypothalamic ERK and CREB phosphorylation in mice that had undergone surgery and thus attenuated the expression of CRH and the hyperactivity of the HPA axis, demonstrating that the ERK/CREB signaling pathway might be involved in EA modulation of HPA axis hyperactivity. Previous research demonstrated that surgery led to ERK5 phosphorylation in the hypothalamus, while EA reduced the secretion of CRH by downregulating the activity of ERK5 (Chen et al., 2015). Besides, EA reduced the number of phosphorylated CREB positive cells in the anterior cingulate cortex (ACC), thus alleviating pain memory (Sun et al., 2015). EA ameliorated memory deficits and alleviated hippocampal synaptic plasticity impairment via the PKA/CREB signaling pathway in rats (Zheng et al., 2016).

We investigated the role of the GluN2A/ERK/CREB signaling pathway in the regulation of HPA axis hyperactivity by EA, provided a new perspective for better understanding the molecular mechanisms of surgery activating the HPA axis, and offered a feasible method for HPA axis hyperactivity regulation. Compared with pharmacological therapy, EA has the advantages of few side effects, good patient tolerance, and immediate effect. The ability of EA to inhibit the GluN2A/ERK/CREB signaling pathway could potentially alleviate HPA axis hyperactivity, although more studies are required to clarify the precise mechanism of EA in regulating the HPA axis.

## Conclusion

Our study demonstrated that surgery enhanced the phosphorylation of the hypothalamic GluN2A/ERK/CREB signaling pathway, thereby aggravating the hyperactivity of the HPA axis. EA intervention at the *Zusanli* and *Sanyinjiao* acupoints alleviated the activation of GluN2A and the downstream ERK/CREB signaling pathway, which downregulated CRH secretion and thus relieved the HPA axis hyperactivity induced by surgery.

## Data Availability Statement

The raw data supporting the conclusions of this article will be made available by the authors, without undue reservation.

## Ethics Statement

The animal study was reviewed and approved by the Animal Care and Use Research Ethical Standards of School of Basic Medical Sciences Fudan University.

## Author Contributions

YW: designed and performed the experiments, prepared the manuscript, and data curation. JH: methodology and prepared the manuscript. JZ: designed and performed experiments, and prepared the manuscript. MZ: technical support, manuscript writing, and editing. MJ: technical support, manuscript writing, and editing. YD: methodology and drafting of the manuscript. ZT: designed and performed the experiments, and funding acquisition. All authors contributed to the article and approved the submitted version.

## Conflict of Interest

The authors declare that the research was conducted in the absence of any commercial or financial relationships that could be construed as a potential conflict of interest.

## Publisher’s Note

All claims expressed in this article are solely those of the authors and do not necessarily represent those of their affiliated organizations, or those of the publisher, the editors and the reviewers. Any product that may be evaluated in this article, or claim that may be made by its manufacturer, is not guaranteed or endorsed by the publisher.
