# Supplementary material for: GluN2A/ERK/CREB Signaling Pathway Involved in Electroacupuncture Regulating Hypothalamic-Pituitary-Adrenal Axis Hyperactivity
Source: Front Neurosci. 2021 Sep 30;15:703044. doi: 10.3389/fnins.2021.703044 (PMC8514998; doi:10.3389/fnins.2021.703044)
Supplement: Supplementary file 1 [file Data_Sheet_1.docx]

Supplementary Material

# Supplementary Figures and Tables


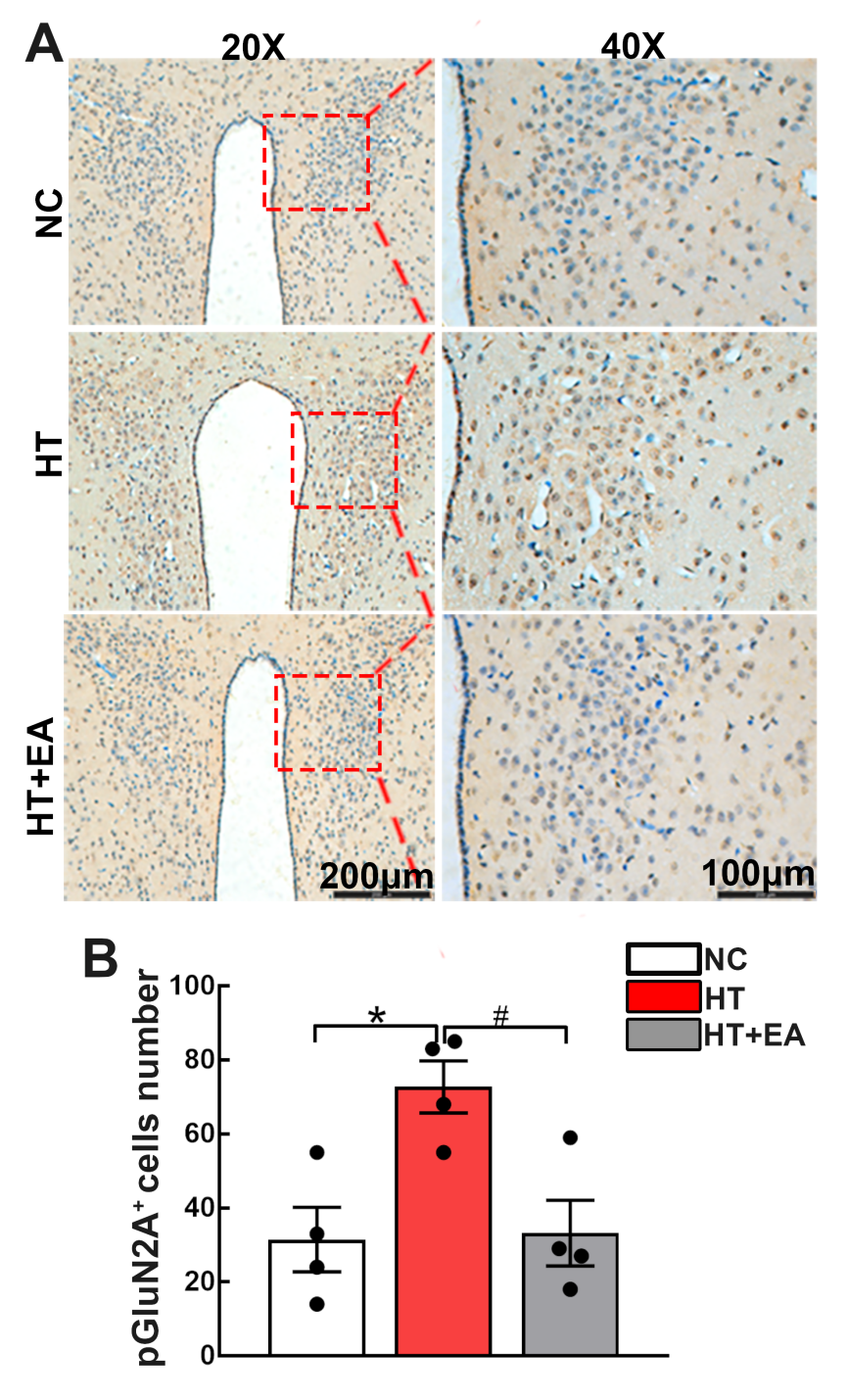


**Supplementary Figure 1.** (A) Representative photomicrographs and (B) quantification for phosphorylated GluN2A-positive cells in the PVN in the NC, HT, and HT+EA groups. (n=4 in each group). Data are expressed as mean ± SEM. *vs. NS group (*p < 0.05, **p < 0.01, ***p < 0.001); #vs. HT group (#p < 0.05, # #p < 0.01, # # #p < 0.001).
